# Supplementary material for: Pharmacological inhibition of TBK1/IKKε blunts immunopathology in a murine model of SARS-CoV-2 infection
Source: Nat Commun. 2023 Sep 18;14:5666. doi: 10.1038/s41467-023-41381-9 (PMC10507085; doi:10.1038/s41467-023-41381-9)

SUPPLEMENTARY INFORMATION

Title: Pharmacological inhibition of TBK1/IKKε blunts immunopathology in a murine model of SARS-CoV-2 infection

Tomalika R. Ullah[1,2], Matt D. Johansen[3], Katherine R. Balka[4], Rebecca L. Ambrose[1,2], Linden J. Gearing[1,2], James Roest[5], Julian P. Vivian[5,6], Sunil Sapkota[1,2], W. Samantha N. Jayasekara[1,2], Daniel S. Wenholz[7,8], Vina R. Aldilla[8], Jun Zeng[9], Stefan Miemczyk[3], Duc H. Nguyen[3], Nicole G. Hansbro[3], Rajan Venkatraman[4], Jung Hee Kang[4], Ee Shan Pang[4], Belinda J. Thomas[1,2,10], Arwaf S. Alharbi[1,2,11], Refaya Rezwan[1,2] Meredith O'Keeffe[4], William A. Donald[8], Julia I. Ellyard[12,13], Wilson Wong[1,2,14], Naresh Kumar[8], Benjamin T. Kile[4,15], Carola G. Vinuesa[12,13,16], Graham E. Kelly[7], Olivier F. Laczka[7], Philip M. Hansbro[3], Dominic De Nardo[4], Michael P. Gantier[1,2*]

*Correspondence to: Michael.gantier@hudson.org.au

This includes:

Supplementary Figs. 1 to 6
Supplementary Table 1 & 2
Supplementary Methods
Uncropped scan of blots from Supplementary Figs.

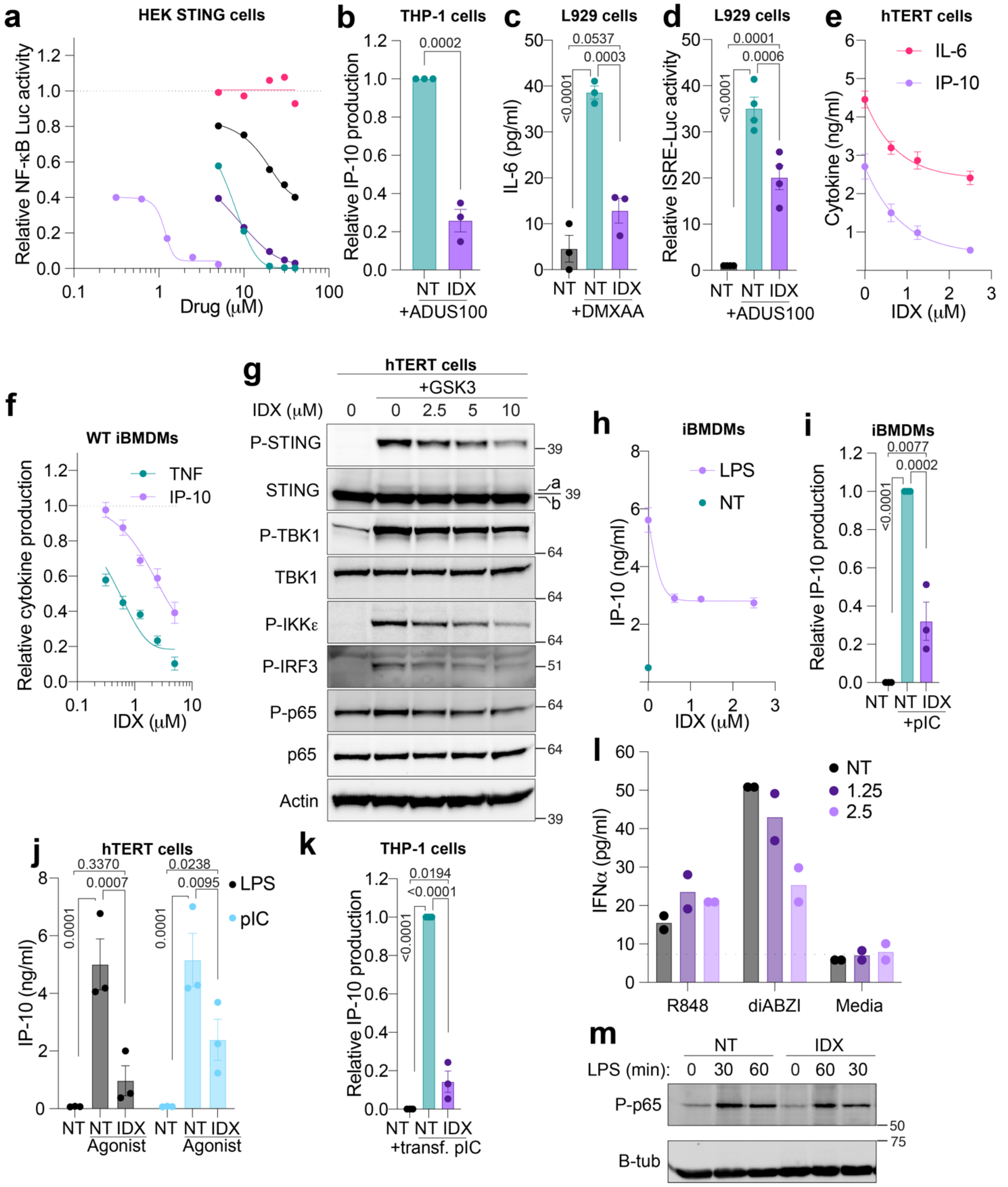

Supplementary Figure 1: IDX inhibits TBK1-dependent IRF3 and NF-κB transcriptional programs. (a) NF-κB luciferase expression in HEK293T mSTING cells over-expressing cGAS treated O/N with the indicated compounds (n=2 independent experiments). (b) IP-10 protein levels in THP-1 monocytes treated with 30 μM ADUS100 for 6 h ± 2.5 μM IDX treatment (n=3 independent experiments). (c, d) IL-6 protein levels (c – n=3 independent experiments) and ISRE-luciferase expression (d – n=4 independent experiments) in L929 cells after 8 h stimulation with STING agonists (20 μg/ml DMXAA or 30 μM ADUS100) with 2.5 μM IDX treatment. (e) IL-6 and IP-10 protein levels in hTERT cells after O/N stimulation with 100 nM GSK3 (STING) (n=3 independent experiments). (f) TNF and IP-10 protein levels after O/N stimulation of iBMDMs with 50 μg/ml DMXAA (STING) and IDX (n=3 independent experiments). (g) Immunoblot of hTERT cells

stimulated with 100 nM GSK3 for 30 min in the presence of the indicated concentrations of IDX [μM] (1 representative blot of 3 independent experiments is shown). Molecular weight markers are shown in kDa. Phosphorylated proteins are indicated with [P]. A doublet of STING bands appears upon STING activation (a corresponds to P-STING). (h) IP-10 protein levels in wild-type iBMDMs after O/N stimulation with 1 μg/ml LPS (TLR4) and IDX (n=3 independent experiments). (i) Relative IP-10 protein levels in wild-type iBMDMs after O/N stimulation with 100 μg/ml polyI:C (TLR3) and 2.5 μM IDX treatment (n=3 independent experiments). (j) IP-10 protein levels in hTERT fibroblasts stimulated O/N with 100 ng/ml LPS (TLR4) or 20 μg/ml poly(I:C) (TLR3), ± 2.5 μM IDX treatment (n=3 independent experiments). (k) IP-10 protein levels in THP-1 monocytes transfected with 1 μg/ml polyI:C (MDA5/RIG-I) ± 2.5 μM IDX treatment O/N (n=3 independent experiments). (l) Splenic plasmacytoid dendritic cells were stimulated overnight with R848 (10 μg/ml – TLR7) or diABZI (0.5 μM - STING) in the presence of 0, 1.25 or 2.5 μM IDX, prior to IFNα quantification in the supernatants by LegendPlex cytometric assay (n=2 independent experiments). (m) Immunoblot of *Tbk1-Ikbkε*^{DKO} iBMDM after 0, 30, and 60 min stimulation with 200 ng/ml LPS (TLR4) and 2.5 μM IDX (1 representative blot of 2 independent experiments is shown). Molecular weight markers are shown in kDa. Phosphorylated proteins are indicated with [P]. (b, c, d, e, f, h, i, j, k) Data are mean ± s.e.m. (b) Unpaired two-tailed t-test is shown. (c, d, i, k) One-way or (j) two-way ANOVA with uncorrected Fisher's LSD (with single pooled variance) multiple comparisons are shown. Exact p values are shown for all comparisons. (a, e, f and h) Non-linear regression analyses are shown. Source data and detailed statistical analyses are provided as a Source Data file.

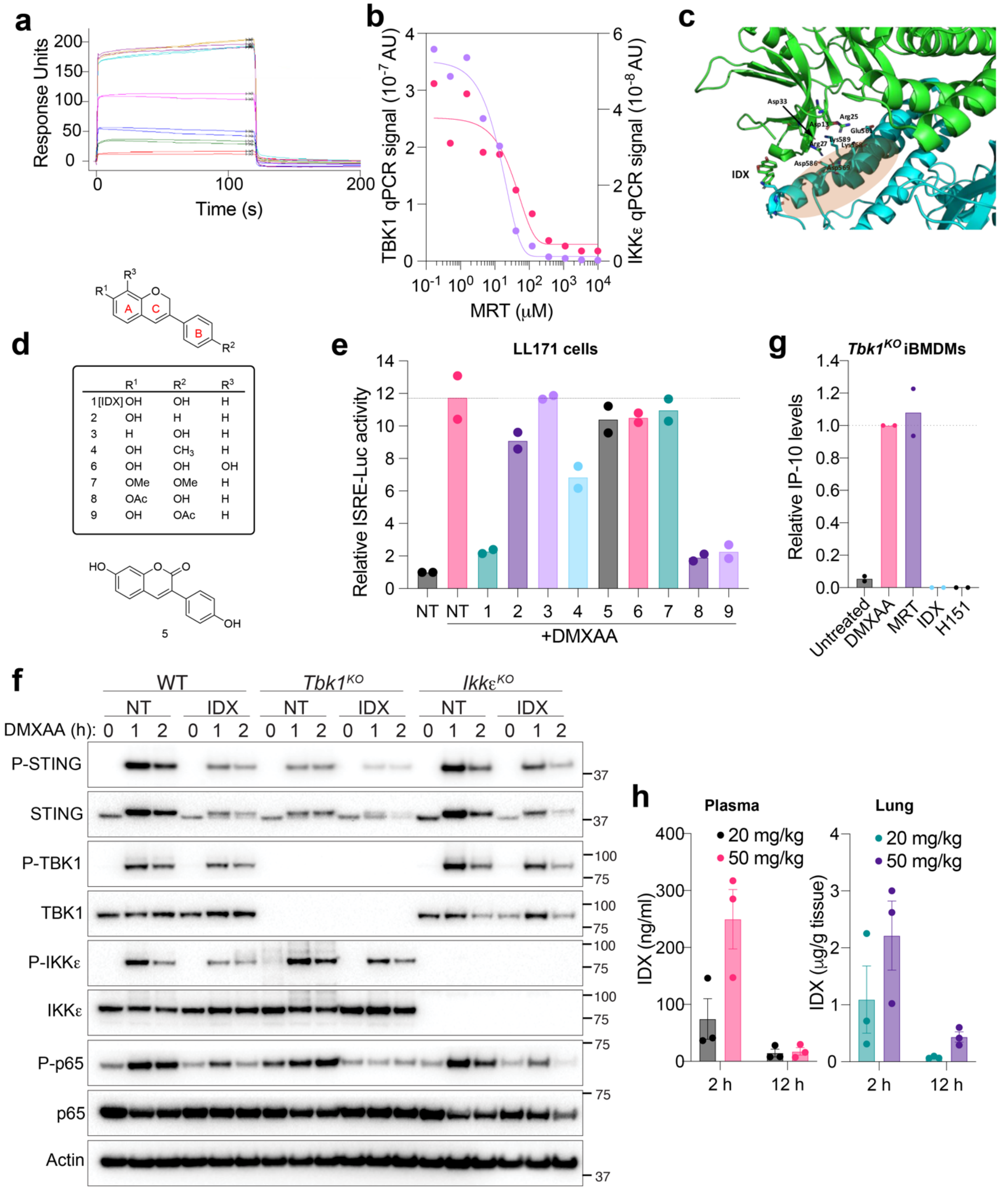

Supplementary Figure 2: Molecular definition of the activity of idronoxil on TBK1 signalling. (a) Representative sensorgrams of TBK1 and IDX SPR (representative of n=3 independent experiments). (b) qPCR amplification signal of kinase concentration after KinomeScan™ assays, for the indicated MRT concentrations (averaged from n=2 technical replicates). (c) IDX interaction with TBK1 dimers in cartoon form, highlighting the residues predicted to form long-range electrostatic interactions between residues of the two TBK1 units (highlighted in green and cyan). (d) Structure of IDX derivatives. (e) ISRE-luciferase expression (n=2 independent experiments) in L929 cells after 8 h stimulation with 20 μg/ml DMXAA (STING) with 2.5 μM of indicated compound. (d, e) The A and B-ring phenol groups of IDX (**1**) are essential for STING inhibition (as observed with **2**, **3** and **4**). Changing the 2H-chromene core to a coumarin core (**5**), addition of phenol to the 8-position of

the A-ring (**6**), or methylation of both A- and B-ring phenol groups (**7**) reduced inhibitory activity. However, selective esterification of either the A- or B-ring phenol group (**8** and **9**, respectively) did not significantly block inhibitory activity. (f) Immunoblot of wild-type, *Tbk1*^{KO} and *Ikbkε*^{KO} iBMDM cells after stimulation with 50 μg/ml DMXAA (STING) and 2.5 μM IDX treatment for the indicated times (1 representative blot of 2 independent experiments is shown). Molecular weight markers are shown in kDa. Phosphorylated proteins are indicated with [P]. (g) Relative IP-10 protein levels in *Tbk1-Ikbkε*^{DKO} iBMDM after O/N stimulation with 50 μg/ml DMXAA (STING) and 1.25 μM of IDX or 100 nM MRT treatment (n=2 independent experiments). (h) IDX levels in mice plasma and lung homogenates at 2 or 12 h post-injection with 20 or 50 mg/kg, determined by LC-MS/MS (n=3 mice per group). (b) Non-linear regression analyses are shown. (h) Data are mean ± s.e.m. Source data are provided as a Source Data file.

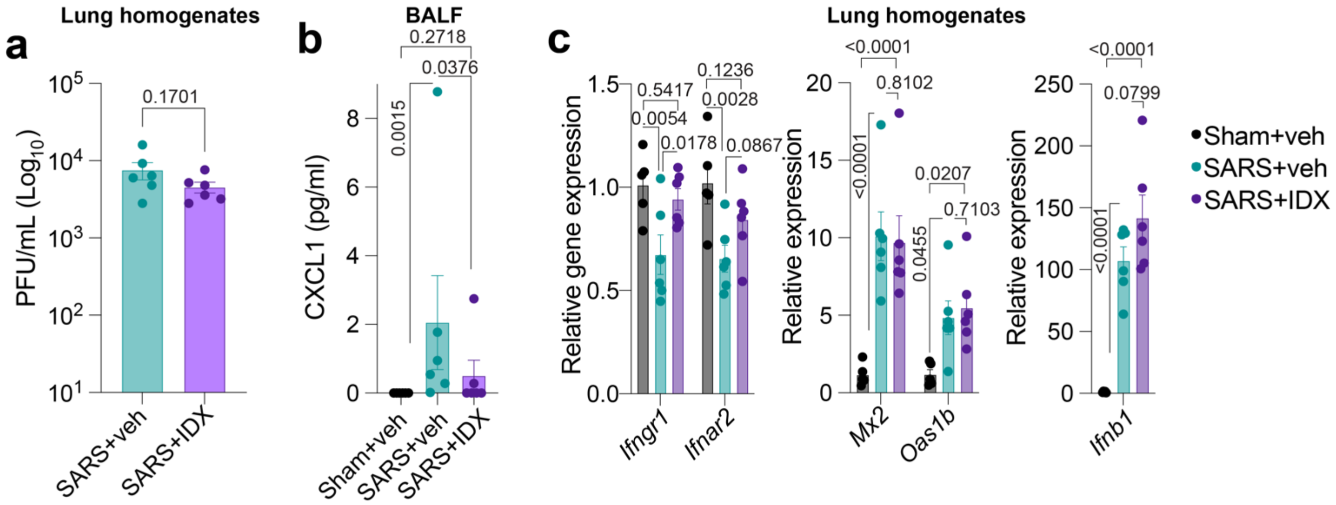

Supplementary Figure 3: Idronoxil does not impact the viral load in the lung. (a) Idronoxil [IDX]/vehicle injections were performed on days 3, 4, and 5, with mice culled on day 6 post-infection. Viral titres in lung homogenates were determined as detailed in Methods (n=6 animals were examined per group in one experiment). Data are mean ± s.e.m. and two-tailed unpaired t-test is shown. Veh is vehicle, SARS is SARS-CoV-2 infected, and Sham is non-infected. (b) CXCL1 protein levels in bronchoalveolar lavage fluid (BALF) samples (n=6 animals were examined per group in one experiment). Data are mean ± s.e.m. and Kruskal-Wallis test with uncorrected Dunn's multiple comparisons are shown. (c) mRNA transcripts in lung homogenates. Expression of indicated genes is shown relative to *Hprt* (n=6 animals were examined per group in one experiment, however qPCR amplification failed for one animal in the Sham+vehicle group). Data are mean ± s.e.m. and one-way (*Ifnb1*) and two-way (*Ifngr1*, *Ifnar2*, *Mx2*, *Oas1b*) ANOVA with uncorrected Fisher's LSD (with single pooled variance) multiple comparisons are shown. Exact p values are shown for all comparisons. Source data and detailed statistical analyses are provided as a Source Data file.

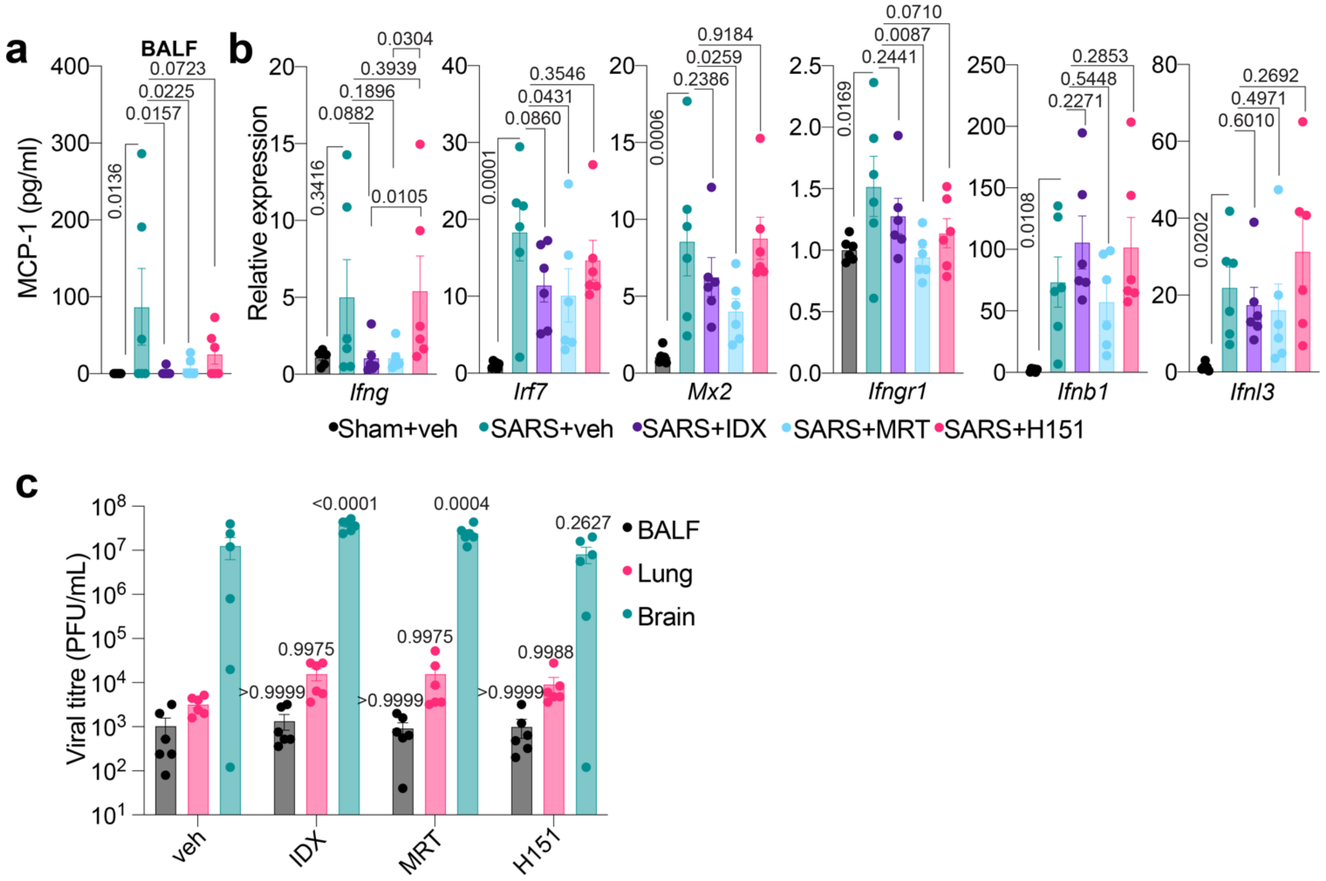

Supplementary Figure 4: TBK1 inhibition limits SARS-CoV-2-driven hyper-inflammation but leads to increased viral load in the brain. (a) Idronoxil [IDX]/ MRT67307 [MRT]/H151/vehicle injections were performed on days 3, 4, and 5, with mice culled on day 5 post-infection (n=6 animals were examined per group in one experiment). MCP-1 quantification in bronchoalveolar lavage fluid (BALF) was performed (n=6 animals were examined per group in one experiment). Data are mean ± s.e.m. and one-way ANOVA with uncorrected Fisher's LSD (with single pooled variance) multiple comparisons are shown. Veh is vehicle, SARS is SARS-CoV-2 infected, and Sham is non-infected. (b) mRNA transcript levels in lung homogenates (n=6 animals were examined per group in one experiment). Expression of indicated genes is shown relative to *Hprt.* Data are mean ± s.e.m. (b [*Ifng*]) Kruskal-Wallis test with uncorrected Dunn's multiple comparisons are shown. (b [*Irf7, Mx2, Ifngr1, Ifnb1, Ifnl3*]) one-way ANOVA with uncorrected Fisher's LSD (with single pooled variance) multiple comparisons are shown. (c) Viral titres in BALF, lung and brain homogenates at day 5 post infection (n=6 animals were examined per group in one experiment). (c) Two-way ANOVA with uncorrected Fisher's LSD (with single pooled variance) multiple comparisons are shown compared to vehicle conditions for each tissue. Source data and detailed statistical analyses are provided as a Source Data file.

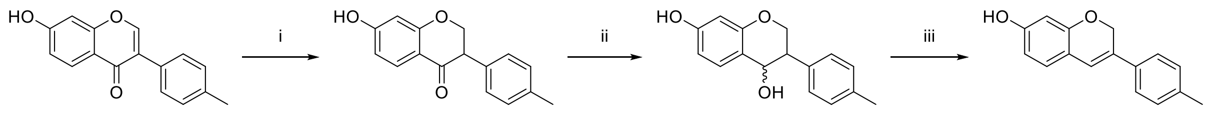

10 11 12 4

Supplementary Figure 5: Reaction Scheme 1. Synthesis of 3-(4-hydroxyphenyl)-6-methyl-2*H*-chromen-7-ol **4 (see Supplementary Methods)**. i) DIBAL-H, -78 °C, 1 h then rt; ii) BH_3 :DMS, THF, 1 h; iii) pTSA, THF, 2 h, 13% yield (over 3 steps).

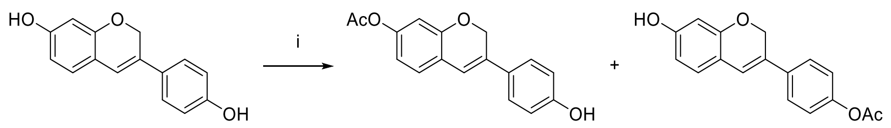

1 8 9

Supplementary Figure 6: Reaction Scheme 2. Synthesis of 3-(4-hydroxyphenyl)-2*H*-chromen-7-yl acetate **8** and 4-(7-hydroxy-2*H*-chromen-3-yl)-phenyl acetate **9 (see Supplementary Methods)**. i) Ac_2O, K_2CO_3, acetone, reflux, o/n, 12% and 11% yields, respectively.

Supplementary Table 1: SARS-CoV-2 Antiviral assay. The percentage of Vero cells infected by SARS-CoV-2 at 24 h post-infection. Eight dilutions of test and control compounds were tested as indicated in the table. Three technical replicates were performed. Untreated infected and untreated uninfected controls were included. Percentage infection was determined as defined in the Methods.

Remdesivir concentration (μM)	Idronoxil concentration (μM)	Idronoxil % infected cells			Remdesivir % infected cells			Untreated % infected cells	Uninfected
20	50	30.75	21.97	29.18	0.16	0.15	0.19	39.25	0.04
6.67	16.67	44.17	33.03	30.88	2	0.33	0.91	19.26	0.03
2.22	5.56	40.66	23.22	28.02	15.16	4.53	16.53	32.77	0.02
0.74	1.85	56.32	60.79	58.12	27.29	18.88	10.1	30.04	0.05
0.25	0.62	39.53	10.77	47.83	13.94	15.42	23.36	21.82	0.02
0.08	0.21	37.79	16.16	24.43	16.75	14.9	52.04	50.71	0.06
0.03	0.07	29.84	31.97	27.82	22.7	40.44	32.08	19.54	0.08
0.01	0.02	32.58	38.33	53.56	29.14	7.93	12.82	42.41	0.01

Supplementary Table 2: KINOMEscan analyses of the inhibition of 468 kinases by IDX. Percentage signal left is provided for the 468 kinases tested with 10000nM IDX, and some of the strongest hits (in red) were validated for Kd determination using the same kinase assays over a range of 11 dilutions of IDX (for PIM2, STK17A, DYRK2, MAP3K19 and TAOK1, similar to what was done for TBK1 and IKKe in Methods section). None of the Kds obtained for these top hits were less than 1 µM supporting a poor inhibitory activity of IDX on these kinases.

Compound Name	DiscoveRx Gene Symbol	Entrez Gene Symbol	Percent Control	Compound Concentration (nM)	Kd (nM)
Idronoxil	PIM2	PIM2	2.6	10000	2300
Idronoxil	DRAK1	STK17A	4.3	10000	1200
Idronoxil	HASPIN	GSG2	5.3	10000	
Idronoxil	TYK2(JH2domain-pseudokinase)	TYK2	8.3	10000	
Idronoxil	DYRK2	DYRK2	11	10000	2700
Idronoxil	ABL1(H396P)-nonphosphorylated	ABL1	12	10000	
Idronoxil	AURKB	AURKB	12	10000	
Idronoxil	YSK4	MAP3K19	12	10000	2200
Idronoxil	KIT(V559D)	KIT	13	10000	
Idronoxil	TAOK1	TAOK1	16	10000	4700
Idronoxil	PDGFRB	PDGFRB	18	10000	
Idronoxil	KIT	KIT	19	10000	
Idronoxil	ABL1(M351T)-phosphorylated	ABL1	21	10000	
Idronoxil	KIT(L576P)	KIT	25	10000	
Idronoxil	PIP5K1C	PIP5K1C	25	10000	
Idronoxil	ABL1(H396P)-phosphorylated	ABL1	26	10000	
Idronoxil	CSF1R	CSF1R	26	10000	
Idronoxil	MAP4K2	MAP4K2	26	10000	
Idronoxil	MYLK4	MYLK4	26	10000	
Idronoxil	DYRK1B	DYRK1B	27	10000	
Idronoxil	PDGFRA	PDGFRA	29	10000	
Idronoxil	ABL1(Q252H)-phosphorylated	ABL1	30	10000	
Idronoxil	ABL1-nonphosphorylated	ABL1	32	10000	
Idronoxil	HIPK3	HIPK3	33	10000	
Idronoxil	ABL1(E255K)-phosphorylated	ABL1	34	10000	
Idronoxil	ABL1(Q252H)-nonphosphorylated	ABL1	34	10000	
Idronoxil	DYRK1A	DYRK1A	34	10000	
Idronoxil	PIM1	PIM1	34	10000	
Idronoxil	VPS34	PIK3C3	34	10000	
Idronoxil	ABL1-phosphorylated	ABL1	35	10000	
Idronoxil	CSNK2A2	CSNK2A2	35	10000	
Idronoxil	ABL1(Y253F)-phosphorylated	ABL1	36	10000	
Idronoxil	CLK4	CLK4	36	10000	
Idronoxil	MINK	MINK1	38	10000	
Idronoxil	DRAK2	STK17B	40	10000	
Idronoxil	PIM3	PIM3	40	10000	

Idronoxil	KIT(V559D,V654A)	KIT	43	10000	
Idronoxil	PHKG2	PHKG2	46	10000	
Idronoxil	HIPK2	HIPK2	47	10000	
Idronoxil	MAP4K4	MAP4K4	47	10000	
Idronoxil	SRC	SRC	47	10000	
Idronoxil	FLT3(ITD,F691L)	FLT3	48	10000	
Idronoxil	CSNK2A1	CSNK2A1	49	10000	
Idronoxil	DAPK3	DAPK3	49	10000	
Idronoxil	ERK8	MAPK15	49	10000	
Idronoxil	PAK2	PAK2	49	10000	
Idronoxil	SGK3	SGK3	49	10000	
Idronoxil	KIT(V559D,T670I)	KIT	50	10000	
Idronoxil	FLT3-autoinhibited	FLT3	51	10000	
Idronoxil	WNK2	WNK2	51	10000	
Idronoxil	JAK3(JH1domain-catalytic)	JAK3	52	10000	
Idronoxil	LRRK2(G2019S)	LRRK2	52	10000	
Idronoxil	MYLK	MYLK	52	10000	
Idronoxil	PIP5K2C	PIP4K2C	52	10000	
Idronoxil	ACVR2B	ACVR2B	53	10000	
Idronoxil	CLK1	CLK1	53	10000	
Idronoxil	DAPK1	DAPK1	53	10000	
Idronoxil	EPHB6	EPHB6	53	10000	
Idronoxil	RPS6KA4(Kin.Dom.2-C-terminal)	RPS6KA4	53	10000	
Idronoxil	TAOK3	TAOK3	53	10000	
Idronoxil	MKNK2	MKNK2	54	10000	
Idronoxil	YANK3	STK32C	54	10000	
Idronoxil	ZAK	ZAK	54	10000	
Idronoxil	LATS2	LATS2	55	10000	
Idronoxil	MKNK1	MKNK1	55	10000	
Idronoxil	DAPK2	DAPK2	56	10000	
Idronoxil	MEK3	MAP2K3	56	10000	
Idronoxil	TNIK	TNIK	56	10000	
Idronoxil	BLK	BLK	57	10000	
Idronoxil	PHKG1	PHKG1	57	10000	
Idronoxil	RSK2(Kin.Dom.1-N-terminal)	RPS6KA3	57	10000	
Idronoxil	CDK8	CDK8	58	10000	
Idronoxil	CSNK1D	CSNK1D	58	10000	
Idronoxil	GAK	GAK	58	10000	
Idronoxil	CDKL3	CDKL3	59	10000	
Idronoxil	PIK3CA(H1047Y)	PIK3CA	59	10000	
Idronoxil	TSSK3	TSSK3	59	10000	
Idronoxil	KIT-autoinhibited	KIT	60	10000	
Idronoxil	LCK	LCK	60	10000	
Idronoxil	NIK	MAP3K14	60	10000	

Idronoxil	PAK1	PAK1	60	10000	
Idronoxil	AAK1	AAK1	61	10000	
Idronoxil	CTK	MATK	61	10000	
Idronoxil	NEK11	NEK11	61	10000	
Idronoxil	VEGFR2	KDR	61	10000	
Idronoxil	CIT	CIT	62	10000	
Idronoxil	MEK5	MAP2K5	62	10000	
Idronoxil	SYK	SYK	62	10000	
Idronoxil	TRKB	NTRK2	62	10000	
Idronoxil	MLK3	MAP3K11	63	10000	
Idronoxil	PIK4CB	PI4KB	63	10000	
Idronoxil	KIT(D816V)	KIT	64	10000	
Idronoxil	MELK	MELK	64	10000	
Idronoxil	CASK	CASK	65	10000	
Idronoxil	EGFR(L858R,T790M)	EGFR	65	10000	
Idronoxil	HIPK1	HIPK1	65	10000	
Idronoxil	IKK-epsilon	IKBKE	65	10000	
Idronoxil	MAPKAPK2	MAPKAPK2	65	10000	
Idronoxil	ACVR2A	ACVR2A	66	10000	
Idronoxil	ANKK1	ANKK1	66	10000	
Idronoxil	BMPR1B	BMPR1B	66	10000	
Idronoxil	EGFR(G719C)	EGFR	66	10000	
Idronoxil	EGFR(T790M)	EGFR	66	10000	
Idronoxil	ERN1	ERN1	66	10000	
Idronoxil	MLCK	MYLK3	66	10000	
Idronoxil	PKN1	PKN1	66	10000	
Idronoxil	TYRO3	TYRO3	66	10000	
Idronoxil	ABL1(F317L)-phosphorylated	ABL1	67	10000	
Idronoxil	CDK7	CDK7	67	10000	
Idronoxil	CHEK2	CHEK2	67	10000	
Idronoxil	CSF1R-autoinhibited	CSF1R	67	10000	
Idronoxil	FLT3(N841I)	FLT3	67	10000	
Idronoxil	NLK	NLK	67	10000	
Idronoxil	VRK2	VRK2	67	10000	
Idronoxil	ALK	ALK	68	10000	
Idronoxil	CAMKK2	CAMKK2	68	10000	
Idronoxil	GRK4	GRK4	68	10000	
Idronoxil	JNK1	MAPK8	68	10000	
Idronoxil	NDR1	STK38	68	10000	
Idronoxil	SNARK	NUAK2	68	10000	
Idronoxil	SRMS	SRMS	68	10000	
Idronoxil	YES	YES1	68	10000	
Idronoxil	CDC2L2	CDC2L2	69	10000	
Idronoxil	CLK2	CLK2	69	10000	

Idronoxil	EPHA1	EPHA1	69	10000	
Idronoxil	HUNK	HUNK	69	10000	
Idronoxil	NEK10	NEK10	69	10000	
Idronoxil	RAF1	RAF1	69	10000	
Idronoxil	RSK1(Kin.Dom.2-C-terminal)	RPS6KA1	69	10000	
Idronoxil	CLK3	CLK3	70	10000	
Idronoxil	MAP4K5	MAP4K5	70	10000	
Idronoxil	PKN2	PKN2	70	10000	
Idronoxil	PRKCI	PRKCI	70	10000	
Idronoxil	EGFR(G719S)	EGFR	71	10000	
Idronoxil	INSR	INSR	71	10000	
Idronoxil	LYN	LYN	71	10000	
Idronoxil	NEK1	NEK1	71	10000	
Idronoxil	PCTK3	CDK18	71	10000	
Idronoxil	PRKG2	PRKG2	71	10000	
Idronoxil	RSK1(Kin.Dom.1-N-terminal)	RPS6KA1	71	10000	
Idronoxil	STK39	STK39	71	10000	
Idronoxil	TLK1	TLK1	71	10000	
Idronoxil	DCAMKL2	DCLK2	73	10000	
Idronoxil	ERBB3	ERBB3	73	10000	
Idronoxil	IKK-beta	IKBKB	73	10000	
Idronoxil	MTOR	MTOR	73	10000	
Idronoxil	PIK3C2G	PIK3C2G	73	10000	
Idronoxil	PRKCQ	PRKCQ	73	10000	
Idronoxil	RIPK4	RIPK4	73	10000	
Idronoxil	ROS1	ROS1	73	10000	
Idronoxil	SLK	SLK	73	10000	
Idronoxil	ACVR1	ACVR1	74	10000	
Idronoxil	AURKC	AURKC	74	10000	
Idronoxil	CAMK1B	PNCK	74	10000	
Idronoxil	CSNK1E	CSNK1E	74	10000	
Idronoxil	EGFR(L747-E749del, A750P)	EGFR	74	10000	
Idronoxil	FGR	FGR	74	10000	
Idronoxil	GSK3A	GSK3A	74	10000	
Idronoxil	PRKG1	PRKG1	74	10000	
Idronoxil	RIPK5	DSTYK	74	10000	
Idronoxil	TYK2(JH1domain-catalytic)	TYK2	74	10000	
Idronoxil	AKT1	AKT1	75	10000	
Idronoxil	BMX	BMX	75	10000	
Idronoxil	DLK	MAP3K12	75	10000	
Idronoxil	HIPK4	HIPK4	75	10000	
Idronoxil	MAPKAPK5	MAPKAPK5	75	10000	
Idronoxil	OSR1	OXSR1	75	10000	
Idronoxil	PIP5K1A	PIP5K1A	75	10000	

Idronoxil	RSK3(Kin.Dom.2-C-terminal)	RPS6KA2	75	10000	
Idronoxil	BRAF	BRAF	76	10000	
Idronoxil	BRSK1	BRSK1	76	10000	
Idronoxil	GRK2	ADRBK1	76	10000	
Idronoxil	MAP3K1	MAP3K1	76	10000	
Idronoxil	MAP3K4	MAP3K4	76	10000	
Idronoxil	PAK4	PAK4	76	10000	
Idronoxil	PKNB(M.tuberculosis)	pknB	76	10000	
Idronoxil	RSK4(Kin.Dom.2-C-terminal)	RPS6KA6	76	10000	
Idronoxil	TNK1	TNK1	76	10000	
Idronoxil	CDKL1	CDKL1	77	10000	
Idronoxil	IRAK3	IRAK3	77	10000	
Idronoxil	PLK1	PLK1	77	10000	
Idronoxil	SGK	SGK1	77	10000	
Idronoxil	YSK1	STK25	77	10000	
Idronoxil	ADCK3	CABC1	78	10000	
Idronoxil	CAMKK1	CAMKK1	78	10000	
Idronoxil	EGFR(L747-T751del,Sins)	EGFR	78	10000	
Idronoxil	EGFR(S752-I759del)	EGFR	78	10000	
Idronoxil	EPHA2	EPHA2	78	10000	
Idronoxil	FER	FER	78	10000	
Idronoxil	FRK	FRK	78	10000	
Idronoxil	HPK1	MAP4K1	78	10000	
Idronoxil	PKAC-alpha	PRKACA	78	10000	
Idronoxil	PKMYT1	PKMYT1	78	10000	
Idronoxil	RIPK1	RIPK1	78	10000	
Idronoxil	STK36	STK36	78	10000	
Idronoxil	TGFBR1	TGFBR1	78	10000	
Idronoxil	ULK3	ULK3	78	10000	
Idronoxil	ZAP70	ZAP70	78	10000	
Idronoxil	ABL2	ABL2	79	10000	
Idronoxil	IRAK4	IRAK4	79	10000	
Idronoxil	MEK2	MAP2K2	79	10000	
Idronoxil	MST3	STK24	79	10000	
Idronoxil	PDPK1	PDPK1	79	10000	
Idronoxil	PIK3CG	PIK3CG	79	10000	
Idronoxil	CAMK1G	CAMK1G	80	10000	
Idronoxil	FGFR2	FGFR2	80	10000	
Idronoxil	FGFR3	FGFR3	80	10000	
Idronoxil	FLT3	FLT3	80	10000	
Idronoxil	IRAK1	IRAK1	80	10000	
Idronoxil	MYLK2	MYLK2	80	10000	
Idronoxil	NEK3	NEK3	80	10000	
Idronoxil	PFCDPK1(P.falciparum)	CDPK1	80	10000	

Idronoxil	PIK3CA(C420R)	PIK3CA	80	10000	
Idronoxil	PLK2	PLK2	80	10000	
Idronoxil	WNK3	WNK3	80	10000	
Idronoxil	ABL1(T315I)-phosphorylated	ABL1	81	10000	
Idronoxil	ADCK4	ADCK4	81	10000	
Idronoxil	IKK-alpha	CHUK	81	10000	
Idronoxil	JAK2(JH1domain-catalytic)	JAK2	81	10000	
Idronoxil	LKB1	STK11	81	10000	
Idronoxil	MAP3K3	MAP3K3	81	10000	
Idronoxil	MET	MET	81	10000	
Idronoxil	RIPK2	RIPK2	81	10000	
Idronoxil	SNRK	SNRK	81	10000	
Idronoxil	CSNK1G2	CSNK1G2	82	10000	
Idronoxil	MLK1	MAP3K9	82	10000	
Idronoxil	MLK2	MAP3K10	82	10000	
Idronoxil	RIOK2	RIOK2	82	10000	
Idronoxil	ROCK2	ROCK2	82	10000	
Idronoxil	CAMK2B	CAMK2B	83	10000	
Idronoxil	EGFR	EGFR	83	10000	
Idronoxil	EGFR(L858R)	EGFR	83	10000	
Idronoxil	HCK	HCK	83	10000	
Idronoxil	MAP3K2	MAP3K2	83	10000	
Idronoxil	PIK3C2B	PIK3C2B	83	10000	
Idronoxil	RET(V804L)	RET	83	10000	
Idronoxil	RPS6KA4(Kin.Dom.1-N-terminal)	RPS6KA4	83	10000	
Idronoxil	TTK	TTK	83	10000	
Idronoxil	WNK1	WNK1	83	10000	
Idronoxil	ABL1(F317L)-nonphosphorylated	ABL1	84	10000	
Idronoxil	AKT3	AKT3	84	10000	
Idronoxil	AMPK-alpha2	PRKAA2	84	10000	
Idronoxil	BRAF(V600E)	BRAF	84	10000	
Idronoxil	BRSK2	BRSK2	84	10000	
Idronoxil	CAMK2D	CAMK2D	84	10000	
Idronoxil	EPHA4	EPHA4	84	10000	
Idronoxil	EPHB1	EPHB1	84	10000	
Idronoxil	FLT3(K663Q)	FLT3	84	10000	
Idronoxil	IGF1R	IGF1R	84	10000	
Idronoxil	LZK	MAP3K13	84	10000	
Idronoxil	MARK2	MARK2	84	10000	
Idronoxil	S6K1	RPS6KB1	84	10000	
Idronoxil	TIE1	TIE1	84	10000	
Idronoxil	WEE1	WEE1	84	10000	
Idronoxil	CAMK2G	CAMK2G	85	10000	
Idronoxil	CDK4-cyclinD3	CDK4	85	10000	

Idronoxil	EGFR(E746-A750del)	EGFR	85	10000	
Idronoxil	FGFR3(G697C)	FGFR3	85	10000	
Idronoxil	GRK3	ADRBK2	85	10000	
Idronoxil	MEK6	MAP2K6	85	10000	
Idronoxil	p38-beta	MAPK11	85	10000	
Idronoxil	PRKD3	PRKD3	85	10000	
Idronoxil	PRKR	EIF2AK2	85	10000	
Idronoxil	RET(M918T)	RET	85	10000	
Idronoxil	ROCK1	ROCK1	85	10000	
Idronoxil	ABL1(F317I)-phosphorylated	ABL1	86	10000	
Idronoxil	BIKE	BMP2K	86	10000	
Idronoxil	CSNK1A1	CSNK1A1	86	10000	
Idronoxil	EPHB2	EPHB2	86	10000	
Idronoxil	MEK1	MAP2K1	86	10000	
Idronoxil	PIK3CD	PIK3CD	86	10000	
Idronoxil	TESK1	TESK1	86	10000	
Idronoxil	TLK2	TLK2	86	10000	
Idronoxil	ACVR1B	ACVR1B	87	10000	
Idronoxil	CAMK2A	CAMK2A	87	10000	
Idronoxil	DDR1	DDR1	87	10000	
Idronoxil	FLT3(D835Y)	FLT3	87	10000	
Idronoxil	SIK2	SIK2	87	10000	
Idronoxil	TSSK1B	TSSK1B	87	10000	
Idronoxil	TXK	TXK	87	10000	
Idronoxil	ASK2	MAP3K6	88	10000	
Idronoxil	CHEK1	CHEK1	88	10000	
Idronoxil	EIF2AK1	EIF2AK1	88	10000	
Idronoxil	ERBB2	ERBB2	88	10000	
Idronoxil	MRCKB	CDC42BPB	88	10000	
Idronoxil	PKAC-beta	PRKACB	88	10000	
Idronoxil	PLK4	PLK4	88	10000	
Idronoxil	PRKX	PRKX	88	10000	
Idronoxil	STK33	STK33	88	10000	
Idronoxil	STK35	STK35	88	10000	
Idronoxil	TRPM6	TRPM6	88	10000	
Idronoxil	ALK(C1156Y)	ALK	89	10000	
Idronoxil	BMPR1A	BMPR1A	89	10000	
Idronoxil	CDK3	CDK3	89	10000	
Idronoxil	EPHA3	EPHA3	89	10000	
Idronoxil	EPHA8	EPHA8	89	10000	
Idronoxil	FLT3(R834Q)	FLT3	89	10000	
Idronoxil	FYN	FYN	89	10000	
Idronoxil	LIMK1	LIMK1	89	10000	
Idronoxil	MET(Y1235D)	MET	89	10000	

Idronoxil	MUSK	MUSK	89	10000	
Idronoxil	NEK5	NEK5	89	10000	
Idronoxil	PIK3CA	PIK3CA	89	10000	
Idronoxil	QSK	KIAA0999	89	10000	
Idronoxil	RSK3(Kin.Dom.1-N-terminal)	RPS6KA2	89	10000	
Idronoxil	TAK1	MAP3K7	89	10000	
Idronoxil	ULK1	ULK1	89	10000	
Idronoxil	AKT2	AKT2	90	10000	
Idronoxil	EPHB4	EPHB4	90	10000	
Idronoxil	ERK5	MAPK7	90	10000	
Idronoxil	FLT3(D835H)	FLT3	90	10000	
Idronoxil	JAK1(JH2domain-pseudokinase)	JAK1	90	10000	
Idronoxil	KIT(D816H)	KIT	90	10000	
Idronoxil	LIMK2	LIMK2	90	10000	
Idronoxil	LRRK2	LRRK2	90	10000	
Idronoxil	MST4	MST4	90	10000	
Idronoxil	p38-alpha	MAPK14	90	10000	
Idronoxil	PIK3CA(H1047L)	PIK3CA	90	10000	
Idronoxil	PRKCH	PRKCH	90	10000	
Idronoxil	TAOK2	TAOK2	90	10000	
Idronoxil	BUB1	BUB1	91	10000	
Idronoxil	CDK9	CDK9	91	10000	
Idronoxil	ERBB4	ERBB4	91	10000	
Idronoxil	KIT(A829P)	KIT	91	10000	
Idronoxil	MARK1	MARK1	91	10000	
Idronoxil	MEK4	MAP2K4	91	10000	
Idronoxil	MKK7	MAP2K7	91	10000	
Idronoxil	MYO3A	MYO3A	91	10000	
Idronoxil	PIKFYVE	PIKFYVE	91	10000	
Idronoxil	SBK1	SBK1	91	10000	
Idronoxil	TNK2	TNK2	91	10000	
Idronoxil	TRKA	NTRK1	91	10000	
Idronoxil	ACVRL1	ACVRL1	92	10000	
Idronoxil	ASK1	MAP3K5	92	10000	
Idronoxil	AURKA	AURKA	92	10000	
Idronoxil	CDK2	CDK2	92	10000	
Idronoxil	DCAMKL3	DCLK3	92	10000	
Idronoxil	DMPK	DMPK	92	10000	
Idronoxil	FGFR1	FGFR1	92	10000	
Idronoxil	FLT3(ITD,D835V)	FLT3	92	10000	
Idronoxil	GSK3B	GSK3B	92	10000	
Idronoxil	MAP4K3	MAP4K3	92	10000	
Idronoxil	NDR2	STK38L	92	10000	
Idronoxil	PRKCE	PRKCE	92	10000	

Idronoxil	RET(V804M)	RET	92	10000	
Idronoxil	DMPK2	CDC42BPG	93	10000	
Idronoxil	EGFR(L861Q)	EGFR	93	10000	
Idronoxil	JAK1(JH1domain-catalytic)	JAK1	93	10000	
Idronoxil	JNK2	MAPK9	93	10000	
Idronoxil	JNK3	MAPK10	93	10000	
Idronoxil	MAST1	MAST1	93	10000	
Idronoxil	MST2	STK3	93	10000	
Idronoxil	NIM1	MGC42105	93	10000	
Idronoxil	PCTK1	CDK16	93	10000	
Idronoxil	TEC	TEC	93	10000	
Idronoxil	ALK(L1196M)	ALK	94	10000	
Idronoxil	CDK4-cyclinD1	CDK4	94	10000	
Idronoxil	ERK1	MAPK3	94	10000	
Idronoxil	FLT3(ITD)	FLT3	94	10000	
Idronoxil	INSRR	INSRR	94	10000	
Idronoxil	PRKD2	PRKD2	94	10000	
Idronoxil	RSK4(Kin.Dom.1-N-terminal)	RPS6KA6	94	10000	
Idronoxil	SRPK3	SRPK3	94	10000	
Idronoxil	YANK1	STK32A	94	10000	
Idronoxil	AXL	AXL	95	10000	
Idronoxil	CSNK1G1	CSNK1G1	95	10000	
Idronoxil	EPHB3	EPHB3	95	10000	
Idronoxil	FES	FES	95	10000	
Idronoxil	FGFR4	FGFR4	95	10000	
Idronoxil	GCN2(Kin.Dom.2,S808G)	EIF2AK4	95	10000	
Idronoxil	LTK	LTK	95	10000	
Idronoxil	MET(M1250T)	MET	95	10000	
Idronoxil	PAK7	PAK7	95	10000	
Idronoxil	PFPK5(P.falciparum)	MAL13P1.27<br>9	95	10000	
Idronoxil	PFTK1	CDK14	95	10000	
Idronoxil	RSK2(Kin.Dom.2-C-terminal)	RPS6KA3	95	10000	
Idronoxil	SGK2	SGK2	95	10000	
Idronoxil	WEE2	WEE2	95	10000	
Idronoxil	WNK4	WNK4	95	10000	
Idronoxil	CAMK1	CAMK1	96	10000	
Idronoxil	CDC2L1	CDK11B	96	10000	
Idronoxil	CSNK1A1L	CSNK1A1L	96	10000	
Idronoxil	EPHA7	EPHA7	96	10000	
Idronoxil	ERK3	MAPK6	96	10000	
Idronoxil	PIK3CA(Q546K)	PIK3CA	96	10000	
Idronoxil	PRKCD	PRKCD	96	10000	
Idronoxil	PRKD1	PRKD1	96	10000	

Idronoxil	RET	RET	96	10000	
Idronoxil	TIE2	TEK	96	10000	
Idronoxil	ABL1(F317I)-nonphosphorylated	ABL1	97	10000	
Idronoxil	ARK5	NUAK1	97	10000	
Idronoxil	BTK	BTK	97	10000	
Idronoxil	CDKL5	CDKL5	97	10000	
Idronoxil	EPHA5	EPHA5	97	10000	
Idronoxil	ITK	ITK	97	10000	
Idronoxil	MARK4	MARK4	97	10000	
Idronoxil	MERTK	MERTK	97	10000	
Idronoxil	MST1	STK4	97	10000	
Idronoxil	PCTK2	CDK17	97	10000	
Idronoxil	SIK	SIK1	97	10000	
Idronoxil	TRKC	NTRK3	97	10000	
Idronoxil	MRCKA	CDC42BPA	98	10000	
Idronoxil	NEK4	NEK4	98	10000	
Idronoxil	PIK3CB	PIK3CB	98	10000	
Idronoxil	PRP4	PRPF4B	98	10000	
Idronoxil	RIOK1	RIOK1	98	10000	
Idronoxil	BMPR2	BMPR2	99	10000	
Idronoxil	CDK11	CDK19	99	10000	
Idronoxil	EGFR(L747-S752del, P753S)	EGFR	99	10000	
Idronoxil	EPHA6	EPHA6	99	10000	
Idronoxil	NEK7	NEK7	99	10000	
Idronoxil	PFTAIRE2	CDK15	99	10000	
Idronoxil	PYK2	PTK2B	99	10000	
Idronoxil	SRPK2	SRPK2	99	10000	
Idronoxil	TBK1	TBK1	99	10000	
Idronoxil	TNNI3K	TNNI3K	99	10000	
Idronoxil	ABL1(T315I)-nonphosphorylated	ABL1	100	10000	
Idronoxil	AMPK-alpha1	PRKAA1	100	10000	
Idronoxil	BRK	PTK6	100	10000	
Idronoxil	CAMK1D	CAMK1D	100	10000	
Idronoxil	CAMK4	CAMK4	100	10000	
Idronoxil	CDC2L5	CDK13	100	10000	
Idronoxil	CDK4	CDK4	100	10000	
Idronoxil	CDK5	CDK5	100	10000	
Idronoxil	CDKL2	CDKL2	100	10000	
Idronoxil	CSK	CSK	100	10000	
Idronoxil	CSNK1G3	CSNK1G3	100	10000	
Idronoxil	DCAMKL1	DCLK1	100	10000	
Idronoxil	DDR2	DDR2	100	10000	
Idronoxil	ERK2	MAPK1	100	10000	
Idronoxil	ERK4	MAPK4	100	10000	

Idronoxil	FAK	PTK2	100	10000	
Idronoxil	FLT1	FLT1	100	10000	
Idronoxil	FLT3(D835V)	FLT3	100	10000	
Idronoxil	FLT4	FLT4	100	10000	
Idronoxil	GRK1	GRK1	100	10000	
Idronoxil	GRK7	GRK7	100	10000	
Idronoxil	ICK	ICK	100	10000	
Idronoxil	LATS1	LATS1	100	10000	
Idronoxil	LOK	STK10	100	10000	
Idronoxil	MAK	MAK	100	10000	
Idronoxil	MAP3K15	MAP3K15	100	10000	
Idronoxil	MARK3	MARK3	100	10000	
Idronoxil	MST1R	MST1R	100	10000	
Idronoxil	MYO3B	MYO3B	100	10000	
Idronoxil	NEK2	NEK2	100	10000	
Idronoxil	NEK6	NEK6	100	10000	
Idronoxil	NEK9	NEK9	100	10000	
Idronoxil	p38-delta	MAPK13	100	10000	
Idronoxil	p38-gamma	MAPK12	100	10000	
Idronoxil	PAK3	PAK3	100	10000	
Idronoxil	PAK6	PAK6	100	10000	
Idronoxil	PIK3CA(E542K)	PIK3CA	100	10000	
Idronoxil	PIK3CA(E545A)	PIK3CA	100	10000	
Idronoxil	PIK3CA(E545K)	PIK3CA	100	10000	
Idronoxil	PIK3CA(I800L)	PIK3CA	100	10000	
Idronoxil	PIK3CA(M1043I)	PIK3CA	100	10000	
Idronoxil	PIP5K2B	PIP4K2B	100	10000	
Idronoxil	PLK3	PLK3	100	10000	
Idronoxil	RIOK3	RIOK3	100	10000	
Idronoxil	RPS6KA5(Kin.Dom.1-N-terminal)	RPS6KA5	100	10000	
Idronoxil	RPS6KA5(Kin.Dom.2-C-terminal)	RPS6KA5	100	10000	
Idronoxil	SgK110	SgK110	100	10000	
Idronoxil	SRPK1	SRPK1	100	10000	
Idronoxil	STK16	STK16	100	10000	
Idronoxil	TGFBR2	TGFBR2	100	10000	
Idronoxil	ULK2	ULK2	100	10000	
Idronoxil	YANK2	STK32B	100	10000	

SUPPLEMENTARY METHODS

Synthesis of idronoxil derivatives

General experimental details:

Yields reported herein refer to purified products (unless specified). Analytical thin-layer chromatography (TLC) was performed on Merck silica gel 60 F_{254} aluminium-backed plates. Compounds were visualised by UV light and/or stained with iodine, ninhydrin or potassium permanganate solution followed by heating. Flash column chromatography was performed on silica gel. ^{1}H-NMR spectra were recorded on a Bruker 400 MHz, Avance II spectrometer with a 5mm DUL (Dual) 13C probe and Bruker 400 MHz, Avance III HD spectrometer with Broad Band Fluorine Observe probe. Chemical shifts (δ) are expressed in parts per million (ppm) with reference to the deuterated solvent peak in which the sample is prepared. Splitting patterns are designated as s (singlet), d (doublet), t (triplet), q (quartet), m (multiplet) and br s (broad singlet).

Experimental procedure:

Compounds **2**, **3**, and **5–7** were synthesised following previously reported procedures.[75-77]

Synthesis of 3-(*p*-tolyl)-2*H*-chromen-7-ol (4) (see Supplementary Figure 5 for reaction scheme). To a stirred solution of 3-(*p*-tolyl)chromane-4,7-diol **12** (0.140 g, crude, 0.542 mmol) in THF (4 ml), *p*-toluenesulfonic acid (0.46 mg, 0.271 mmol) was added. The reaction mixture was stirred for 2 h at room temperature (rt). Progress of reaction was monitored by TLC, which showed consumption of starting material. The reaction mixture was quenched with aqueous $NaHCO_3$ (5 ml) and extracted with ethyl acetate (3 × 10 ml). The combined organic layer was washed with water (25 ml) and brine (25 ml) solution and dried over anhydrous Na_2SO_4, filtered and concentrated under vacuum to produce the crude compound. The crude compound was purified by prep HPLC purification (2 mM ammonium acetate buffer and acetonitrile) to yield 3-(*p*-tolyl)-2*H*-chromen-7-ol **4** (22 mg, 13% over

3 steps) as an off-white solid. LCMS: 237.01 [M-H]$^-$. ^{1}H-NMR (400 MHz; DMSO-d_6) : δ 9.57 (s, 1H, OH), 7.40 (d, J = 8.2 Hz, 2H, ArH), 7.20 (d, J = 8.2 Hz, 2H, ArH), 6.98 (d, J = 8.2 Hz, 1H, ArH), 6.90 (s, 1H, ArH), 6.35–6.32 (dd, J^1 = 8.2, J^2 = 2.2 Hz, 1H, ArH), 6.24 (d, J = 2.2 Hz, 1H, C=CH-), 5.05 (s, 2H, CH_2), 2.30 (s, 3H, CH_3).

Synthesis of 3-(4-hydroxyphenyl)-2*H*-chromen-7-yl acetate (8) (see Supplementary Figure 6 for reaction scheme). To a stirred solution of 3-(4-hydroxyphenyl)-2*H*-chromen-7-ol **1** (15.0 g, 62.4 mmol) in acetone (450 ml) was added potassium carbonate (17.3 g, 125 mmol) followed by acetic anhydride (5.73 g, 56.1 mmol) at room temperature. The reaction mixture was stirred O/N at 58 °C. Progress of reaction was monitored by TLC. The reaction mass was concentrated under reduced pressure and crude mass was purified by flash column chromatography (SFC) (biotage) using 100% DCM to obtain mixture of **8** and **9** (5.5 g). Further purification using supercritical fluid chromatography (CHIRALCEL OJ-H (250 × 21 mm), 5 µm, isocratic, 60% CO_2 and 40% MeOH, RT: 6.6 min for Peak-1, 9.9 min for Peak-2 at 215 nm to obtain 3-(4-hydroxyphenyl)-2*H*-chromen-7-yl acetate **8** (2.1 g, 12%) as an off-white solid from Peak-1. ^{1}H-NMR (400 MHz; DMSO-d_6): δ 9.69 (s, 1H, OH), 7.41–7.38 (m, 2H, ArH), 7.15 (d, J = 8.2 Hz, 1H, ArH), 6.88 (s, 1H, ArH), 6.82–6.78 (m, 2H, ArH), 6.65 (dd, J^1 = 8.1, J^2 = 2.2 Hz, 1H, ArH), 6.62 (d, J = 2.1 Hz, 1H, C=CH-), 5.08 (s, 2H, CH_2), 2.19 (s, 3H, CH_3). LCMS: 281.09 [M-H]$^-$.

Synthesis of 4-(7-hydroxy-2*H*-chromen-3-yl)phenyl acetate (9) (see Supplementary Figure 6 for reaction scheme). We obtained 4-(7-hydroxy-2*H*-chromen-3-yl)phenyl acetate **9** (1.9 g, 11%) as an off-white solid from Peak-2 in SFC purification of 3-(4-hydroxyphenyl)-2H-chromen-7-yl acetate **8**. ^{1}H-NMR (400 MHz; DMSO-d_6): δ 9.63 (s, 1H, OH), 7.55–7.52 (m, 2H, ArH), 7.15–7.13 (m, 2H, ArH), 6.99 (d, J = 8.2 Hz, 1H, ArH), 6.95 (s, 1H, ArH), 6.35 (dd, J^1 = 8.1, J^2 = 2.1 Hz, 1H, ArH), 6.26 (d, J = 2.0 Hz, 1H, C=CH-), 5.08 (s, 2H, CH_2), 2.27 (s, 3H, CH_3). LCMS: 281.06 [M-H]$^-$.

Synthesis of 7-hydroxy-3-(*p*-tolyl)chroman-4-one (11) (see Supplementary Figure 5 for reaction scheme). To a stirred solution of 7-hydroxy-3-(*p*-tolyl)-4*H*-chromen-4-one **10** (0.180 g, 0.714 mmol) in THF (3 ml), DIBAL (2.14 ml, 2.14 mmol, 1M in toluene) was added drop wise at -78 °C. The reaction mixture was stirred for 1 h at -78 °C then allowed to warm to rt. Progress of reaction was monitored by TLC, which showed consumption of starting material. The reaction mixture was quenched with aqueous 1N HCl (3 ml) and extracted with ethyl acetate (3 × 10 ml). The combined organic layer was washed with water (25 ml) and brine (25 ml) solution. The organic layer was dried over anhydrous Na_2SO_4, filtered and concentrated under vacuum to obtain crude 7-hydroxy-3-(*p*-tolyl)chroman-4-one **11** (160 mg, crude) as an off white-solid, which was carried onto the next step without further purification. LCMS: 253.06 $[M-H]^-$

Synthesis of 3-(*p*-tolyl)chromane-4,7-diol (12) (see Supplementary Figure 5 for reaction scheme). To a stirred solution of 7-hydroxy-3-(*p*-tolyl)chroman-4-one **11** (0.160 g, crude, 0.630 mmol) in THF (4 ml) and borane dimethyl sulfide (0.141 ml, 1.88 mmol) was added drop wise at 0 °C. The reaction mixture was stirred at rt for 1h. Progress of reaction was monitored by TLC, which showed consumption of starting material. The reaction mixture was quenched with aqueous 2N HCl (3 ml) and extracted with ethyl acetate (3 × 10 ml). The combined organic layer was washed with water (25 ml) and brine (25 ml) solution. The organic layer was dried over anhydrous Na_2SO_4, filtered and concentrated under vacuum to yield crude 3-(*p*-tolyl)chromane-4,7-diol **12** (0.140 g, crude) as an off-white solid, which was carried onto the next step without further purification. LCMS: 257.18 $[M+H]^+$

NMR Spectra

^{1}H-NMR spectrum of 3-(*p*-tolyl)-2*H*-chromen-7-ol (4).

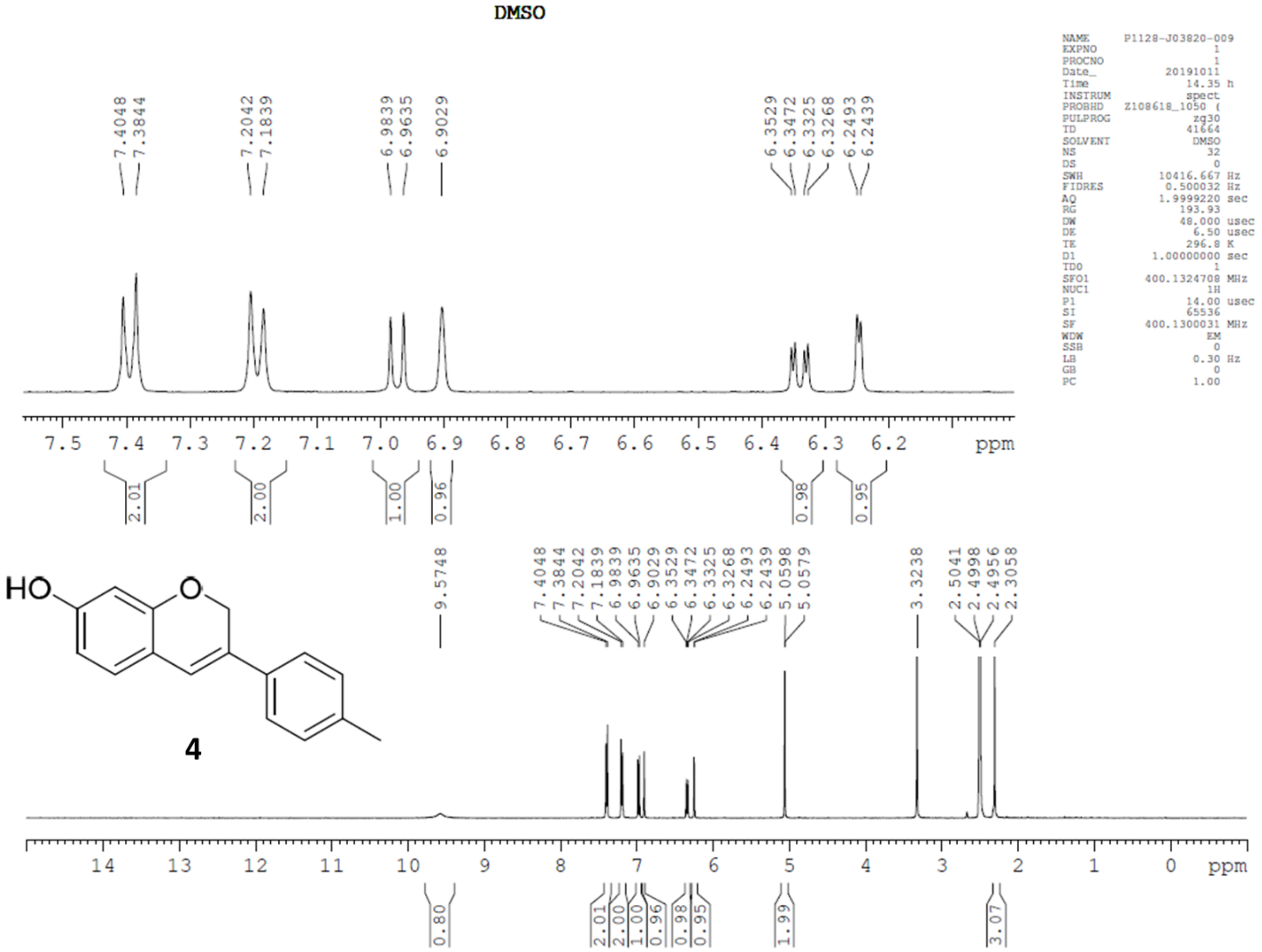

^{1}H-NMR spectrum of 3-(4-hydroxyphenyl)-2*H*-chromen-7-yl acetate (8).

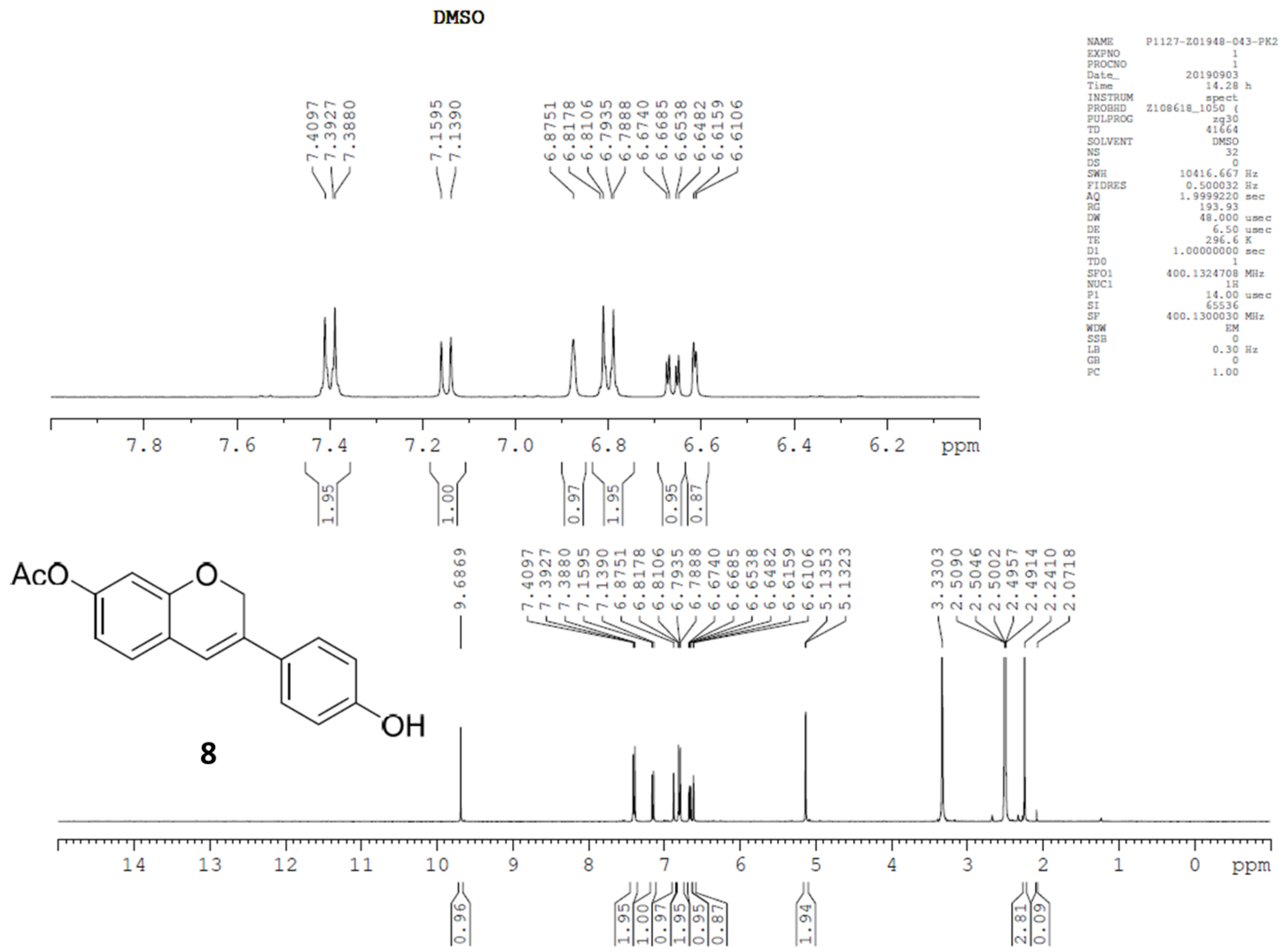

NOE spectrum of 3-(4-hydroxyphenyl)-2*H*-chromen-7-yl acetate (8).

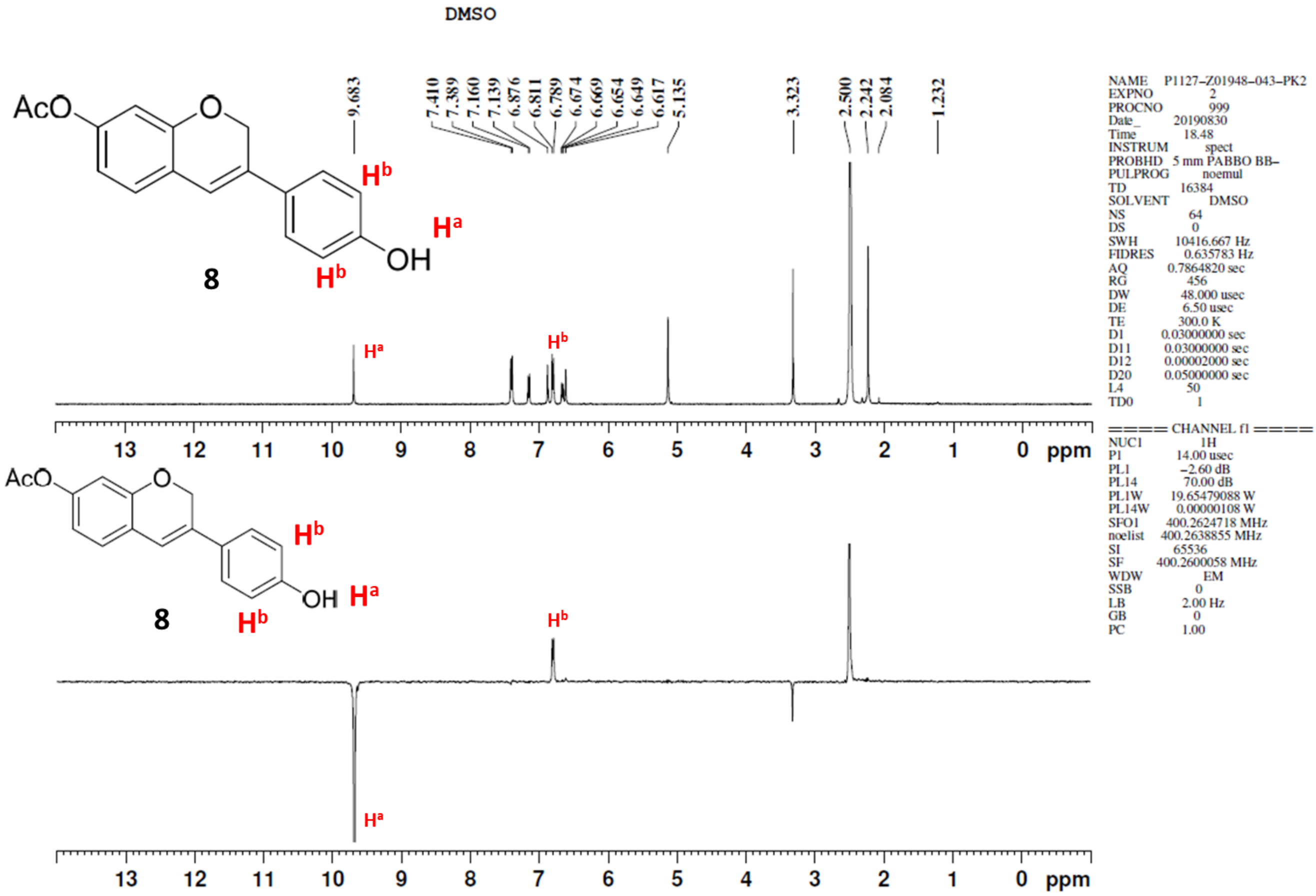

^{1}H-NMR spectrum of 4-(7-hydroxy-2*H*-chromen-3-yl)phenyl acetate (9).

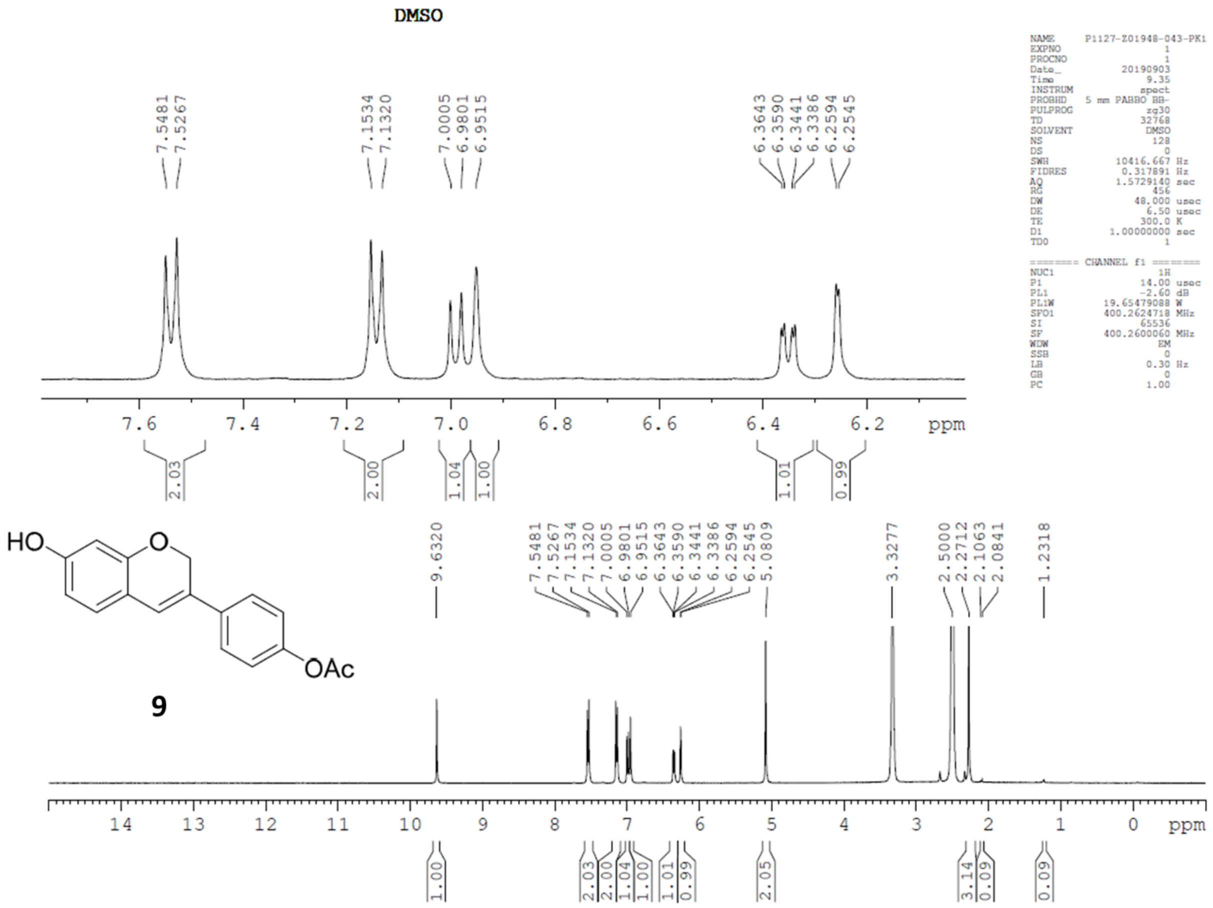

Uncropped scans

Supplementary Figure 1g

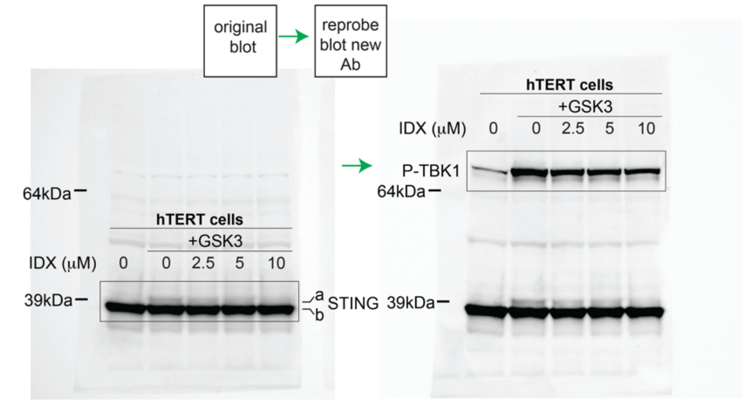

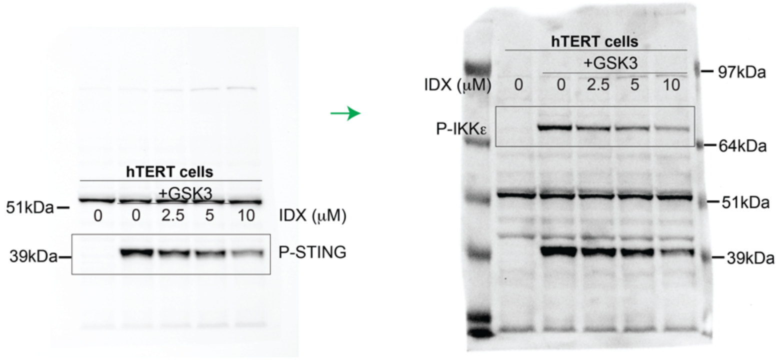

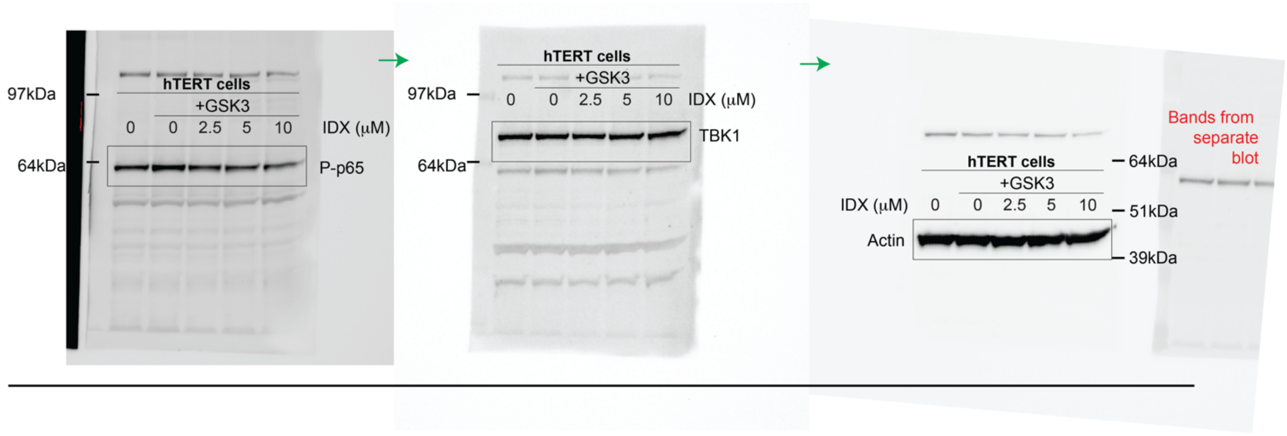

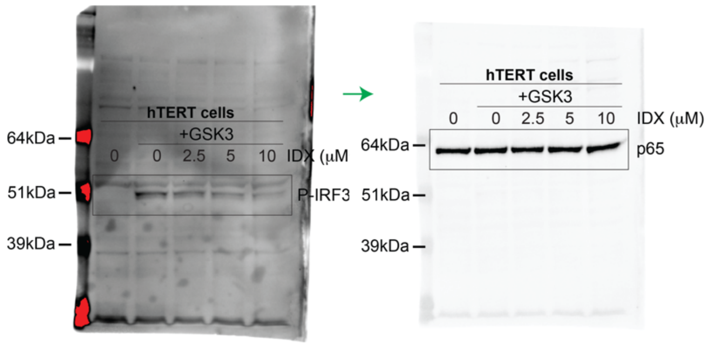

Supplementary Figure 1m

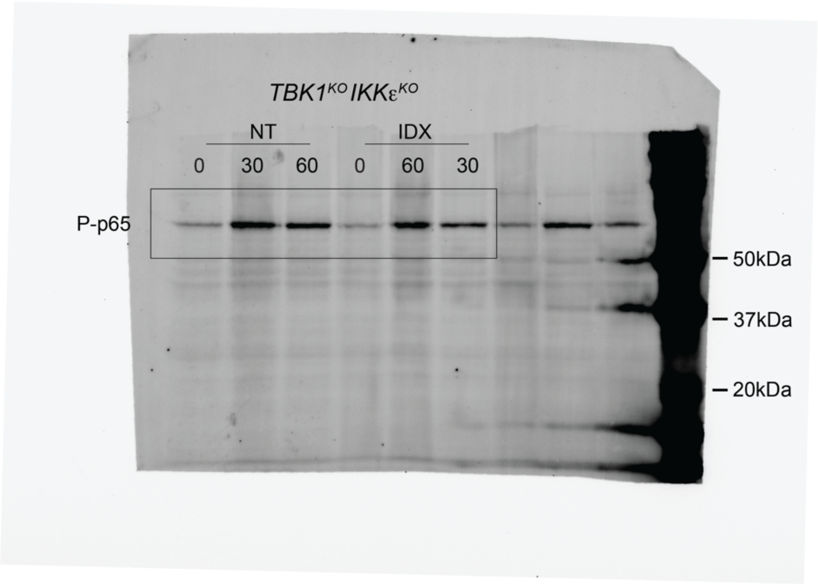

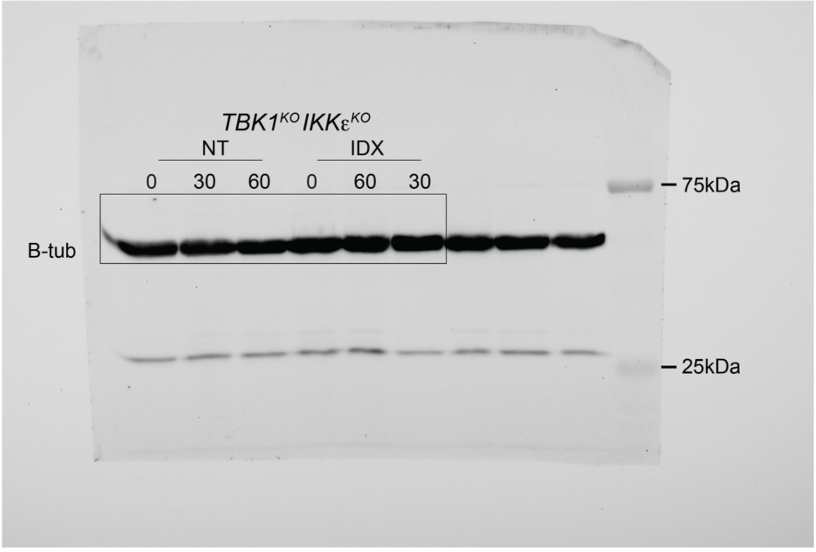

Supplementary Figure 2f

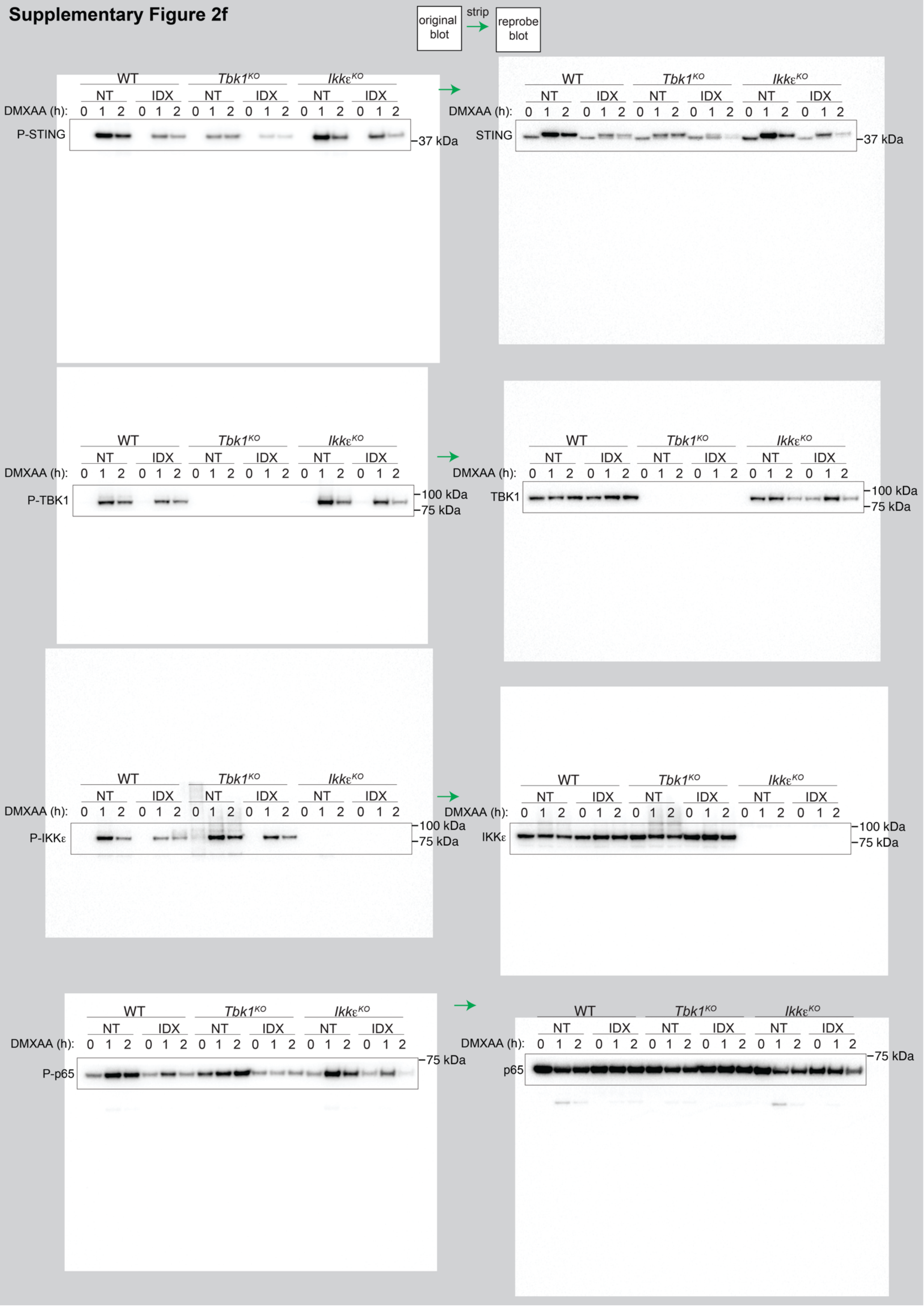

Supplementary Figure 2f

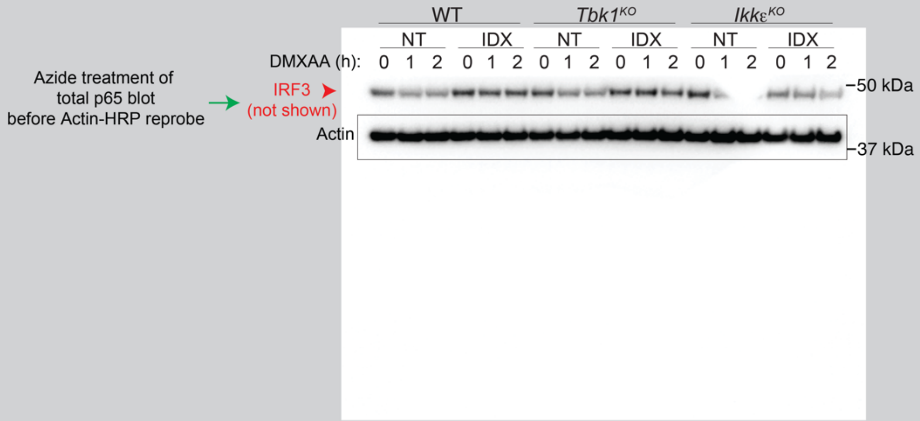

Supplement: Supplementary file 1 — Supplementary Information [file 41467_2023_41381_MOESM1_ESM.pdf]
