# Supplementary material for: Pharmacological inhibition of TBK1/IKKε blunts immunopathology in a murine model of SARS-CoV-2 infection
Source: Nat Commun. 2023 Sep 18;14:5666. doi: 10.1038/s41467-023-41381-9 (PMC10507085; doi:10.1038/s41467-023-41381-9)
Supplement: Supplementary file 3 — Reporting Summary [file 41467_2023_41381_MOESM3_ESM.pdf]

Corresponding author(s): Michael Gantier

Last updated by author(s): 17/08/2023

Reporting Summary

Nature Portfolio wishes to improve the reproducibility of the work that we publish. This form provides structure for consistency and transparency in reporting. For further information on Nature Portfolio policies, see our Editorial Policies and the Editorial Policy Checklist.

Statistics

For all statistical analyses, confirm that the following items are present in the figure legend, table legend, main text, or Methods section.

n/a	Confirmed	
☐	☒	The exact sample size (*n*) for each experimental group/condition, given as a discrete number and unit of measurement
☐	☒	A statement on whether measurements were taken from distinct samples or whether the same sample was measured repeatedly
☐	☒	The statistical test(s) used AND whether they are one- or two-sided<br>*Only common tests should be described solely by name; describe more complex techniques in the Methods section.*
☒	☐	A description of all covariates tested
☐	☒	A description of any assumptions or corrections, such as tests of normality and adjustment for multiple comparisons
☐	☒	A full description of the statistical parameters including central tendency (e.g. means) or other basic estimates (e.g. regression coefficient) AND variation (e.g. standard deviation) or associated estimates of uncertainty (e.g. confidence intervals)
☐	☒	For null hypothesis testing, the test statistic (e.g. *F*, *t*, *r*) with confidence intervals, effect sizes, degrees of freedom and *P* value noted<br>*Give P values as exact values whenever suitable.*
☒	☐	For Bayesian analysis, information on the choice of priors and Markov chain Monte Carlo settings
☒	☐	For hierarchical and complex designs, identification of the appropriate level for tests and full reporting of outcomes
☒	☐	Estimates of effect sizes (e.g. Cohen's *d*, Pearson's *r*), indicating how they were calculated

Our web collection on statistics for biologists contains articles on many of the points above.

Software and code

Policy information about availability of computer code

Data collection	For Western Blot, acquisition of images was conducted with Image Lab 6.1 (Bio-Rad). For SPR analyses the Biacore T200 Evaluation Software Version 3.2 (Cytiva) was used. For ELISA and luminescence collection with Fluostar OPTIMA the OPTIMA-Control v2.2R2 software was used.
Data analysis	For RNA sequencing analyses: Interferome (http://interferome.its.monash.edu.au/interferome/home.jspx)<br>Dragen BCLConvert (v3.7.4) for base calling<br>cutadapt (v2.10) – read demultiplexing for deposition<br>R (v4.1.0) – data analysis with the following packages:<br>scPipe package (v1.14.0) – read processing, demultiplexing, gene counting<br>Rsubread package (v2.6.1) – read alignment<br>biomaRt package (v2.48.2) – gene annotation<br>edgeR package (v3.34.0) – count filtering, normalisation, linear model fitting<br>limma package (v3.48.3) – differential gene expression, gene set testing<br>msigdbr package (v7.4.1) – gene set collections<br>TopKLists package (v1.0.7) - rank combination<br>pheatmap package (v1.0.12) – visualisation<br><br>RTqPCR: QuantStudio Real Time PCR Software v1.7.2 and CFX Maestro Software 2.1.<br>MARS Data analysis software 3.01R2 (BMG Labtech) was used for luminescence and absorbance analyses.<br>Statistical analyses were carried out using Prism 9 (GraphPad Software Inc.)<br>Molecular docking : quCBit (MedChemSoft Solutions, version 2019)

For Western Blot, analyses were conducted with Image Lab 6.1 (Bio-Rad)
ImageJ 1.53 was used to analyse histological microscopy images.

For manuscripts utilizing custom algorithms or software that are central to the research but not yet described in published literature, software must be made available to editors and reviewers. We strongly encourage code deposition in a community repository (e.g. GitHub). See the Nature Portfolio guidelines for submitting code & software for further information.

Data

Policy information about availability of data

All manuscripts must include a data availability statement. This statement should provide the following information, where applicable:

- Accession codes, unique identifiers, or web links for publicly available datasets
- A description of any restrictions on data availability
- For clinical datasets or third party data, please ensure that the statement adheres to our policy

RNA sequencing data has been deposited in the NCBI Gene Expression Omnibus (GEO) with accession GSE193353 (https://www.ncbi.nlm.nih.gov/geo/query/acc.cgi?acc=GSE193353). The interferome v2 database is available at http://interferome.its.monash.edu.au/interferome/home.jspx. Uncropped and unprocessed scans of the representative blots shown in the Figures are available in the Source Data file or at the end of the Supplementary information file. Source data for each line graphs and bar charts are available in Source Data file.

Field-specific reporting

Please select the one below that is the best fit for your research. If you are not sure, read the appropriate sections before making your selection.

- [x] Life sciences
- [ ] Behavioural & social sciences
- [ ] Ecological, evolutionary & environmental sciences

For a reference copy of the document with all sections, see nature.com/documents/nr-reporting-summary-flat.pdf

Life sciences study design

All studies must disclose on these points even when the disclosure is negative.

Sample size	For SARS-CoV-2 mouse infection experiments, we used an n=6/group (based on published studies in the same model PMID: 32839612). This is justified based on feasibility of performing large mouse infection experiments under BSL3 conditions, where capacity is largely reduced. This is the required number of mice to achieve statistical power in all experiments performed. For IDX PK studies in Supp Figure 2h, we used 3 mice per group as this is the minimum required for these analyses. For cell based experiments all were conducted at least 2 independent times in biological replicates (unless otherwise stated). For BMM experiments from TREX1 mutant mice, 3 different mice were tested.
Data exclusions	In the lung cytokine analysis in Fig 3g, there was 1 mouse in the SARS IDX group that was a statistical outlier when a Grubb's test was performed. This sample was approximately 10-fold higher than all other samples in the analysis irrespective of group and therefore was removed from all cytokine analysis displayed in the manuscript.<br>Similarly, for For Fig 4e (CXCL1) and the neutrophil data in Fig 4b, clear outliers (no more than one per group) were identified by ROUT analyses (Q=1) and removed from these specific graphs.<br>For RTqPCR analyses of Fig. 3h and Ext. Fig 3c, one sample from one Sham+Veh mouse gave too little RNA (Cq value for Hprt for this sample was >37), and was not included in further analyses.
Replication	In vitro experiments were all reliably reproduced a minimum of two independent times - except for the Kinase analyses in Figure 2C and Suppl Figure 2B/Supplementary Table 2, and SARS-CoV-2 infections in Vero cells (Supplementary Table 1) which were only conducted once in replicates.
Randomization	Mice were randomly allocated to their respective groups for SARS-CoV-2 infection and treatment regimes prior to the experiment commencing. Mice were also randomly allocated to their group for the IDX PK studies. For preparation of primary splenic pDCs and bone marrow derived macrophages from TREX1 mutant mice, mice from the same genotype and same age were used.
Blinding	Blinding was not feasible in the SARS-CoV-2 mouse infection experiments due to multiple drug administrations required and feasibility of sample processing and retrieval from BSL3 facilities. For PK studies the analyses were not blinded as this was not feasible given the different preparation of the IDX solutions (made fresh) used here.

Reporting for specific materials, systems and methods

We require information from authors about some types of materials, experimental systems and methods used in many studies. Here, indicate whether each material, system or method listed is relevant to your study. If you are not sure if a list item applies to your research, read the appropriate section before selecting a response.

Materials & experimental systems

n/a	Involved in the study
[ ]	[x] Antibodies
[ ]	[x] Eukaryotic cell lines
[x]	[ ] Palaeontology and archaeology
[ ]	[x] Animals and other organisms
[x]	[ ] Human research participants
[x]	[ ] Clinical data
[x]	[ ] Dual use research of concern

Methods

n/a	Involved in the study
[x]	[ ] ChIP-seq
[x]	[ ] Flow cytometry
[x]	[ ] MRI-based neuroimaging

Antibodies

Antibodies used

The following antibodies were used:
Rabbit monoclonal anti-STING (D2P2F) antibody Cell Signaling Technology Cat# 13647 (Human, Mouse), used at 1:1000 dilution [RRID:AB_2732796]
Rabbit monoclonal anti-P-STING Ser365 (D8F4W) antibody Cell Signaling Technology Cat# 72971 (Mouse), used at 1:500 dilution [RRID:AB_2799831]
Rabbit monoclonal anti-P-STING (Ser366) (D7C3S) antibody Cell Signaling Technology Cat# 19781 (Human), used at 1:500 dilution [RRID:AB_2737062]
Rabbit polyclonal anti-TBK1 antibody Cell Signaling Technology Cat# 3013 (Human, Mouse), used at 1:1000 dilution [RRID:AB_2199749]
Rabbit monoclonal antibody anti-P-TBK1 Ser172 (D52C2) Cell Signaling Technology Cat# 5483 (Human, Mouse), used at 1:500 dilution [RRID:AB_10693472]
Rabbit monoclonal anti-IKKε (D61F9) antibody Cell Signaling Technology Cat# 3416 (Mouse), used at 1:1000 dilution [RRID:AB_1264180]
Rabbit monoclonal anti-P-IKKε (Ser172) (D1B7) Cell Signaling Technology Cat#8766 (Human, Mouse), used at 1:500 dilution [RRID:AB_2737061]
Rabbit Polyclonal anti-IKKε Antibody Cell Signaling Technology Cat#2690 (Human), used at 1:1000 dilution [RRID:AB_915926]
Rabbit monoclonal anti-IRF3 (D83B9) antibody Cell Signaling Technology Cat# 4302 (Human, Mouse), used at 1:1000 dilution [RRID:AB_1904036]
Rabbit monoclonal anti-P-IRF3 Ser396 (4D4G) antibody Cell Signaling Technology Cat# 4947 (Human, Mouse), used at 1:500 dilution [RRID:AB_823547]
Rabbit monoclonal anti-NF-kB p65 (C22B4) antibody Cell Signaling Technology Cat# 4764 (Human, Mouse), used at 1:1000 dilution [RRID:AB_823578]
Rabbit monoclonal anti-NF-kB P-p65 Ser536 (93H1) antibody Cell Signaling Technology Cat# 3033 (Human, Mouse), used at 1:500 dilution [RRID:AB_331284]
Mouse monoclonal anti-beta ACTIN, HRP (AC-15) Abcam Cat# Ab49900 (Human, Mouse), used at 1:10,000 dilution [RRID:AB_867494]
Mouse monoclonal anti-GFP antibody (3E6) Thermo Scientific #A-11120, used at 1:1000 dilution (IB) or 1 µg (IP) [RRID:AB_221568]
Peroxidase-AffiniPure Goat Anti-Rabbit IgG (H+L) antibody Jackson ImmunoResearch Labs Cat# 111-035-003, used at 1:10,000 dilution [RRID:AB_2313567]
Goat anti-Mouse IgG (H+L) Highly Cross-Adsorbed Secondary Antibody, HRP Thermo Fisher Scientific Cat# A16078, used at 1:10,00 dilution [RRID:AB_2534751]
Peroxidase-AffiniPure F(ab')2 Fragment Donkey Anti-Rabbit IgG (H+L) antibody Jackson ImmunoResearch Labs Cat# 711-036-152, used at 1:20,00 dilution [RRID:AB_2340590]

Validation

Antibodies were used according to the validated listed by the manufacturer's. Antibody Registry (RRID) numbers are provided above.

Eukaryotic cell lines

Policy information about cell lines

Cell line source(s)

HEK-cGASlow cells, HEK-STING cells, L929 cells expressing an IFN-stimulated response element were reported in PMID: 24077100
WT, StingKO, Tbk1KO, IkkeKO, and Tbk1KO/IkkeKO iBMDMs were reported in PMID: 32268090
BJ-5ta hTERT foreskin fibroblasts were obtained from ATCC (#RL4001)
p125HEK were reported in PMID: 29572905
HEK293T GFP were reported in PMID:20125126
WT THP-1 cells were reported in PMID: 34057477
HEK293 TLR3 were from Invivogen (hkb-htlr3)
Vero cells were from Creative Biolabs (#CAR-STC-ZP39).

Authentication

None of the non-commercial cells lines were authenticated.

Mycoplasma contamination	The cells were routinely tested for mycoplasma by PCR and only used when negative.
Commonly misidentified lines (See ICLAC register)	We did not use any misidentified cell lines.

Animals and other organisms

Policy information about studies involving animals; ARRIVE guidelines recommended for reporting animal research

Laboratory animals	B6.Cg-Tg(K18-ACE2)2Prlmn/J. Female mice (~8 weeks of age) were used for SARS-CoV-2 mouse experiments. It has been reported that male and female K18-hACE2 mice have similar lethality and disease severity at 1000 PFU (Yinda et al. 2022 Plos Path). As such, while sex of mice was a consideration in the design of our study we reasoned that this would have no impact on the outcome of the findings in this manuscript. Given the large numbers of mice involved in performing the SARS-CoV-2 experiments, and the limited capacity of our BSL3 facility, it was not feasible to duplicate these experiments in male mice. 8-week old male BALB/c nude mice were used for IDX tissue distribution studies, however mice gender is not expected to impact the bio-distribution of IDX.<br>For ex vivo experiments, C57BL/6J wild type mice, at 6-8 weeks were used for splenic DC purification. 10-week old TREX1-mutant male mice were used for BMM purifications. Gender was not accounted for in these ex-vivo studies since the analyses assessed treated versus non treated cells from the same animals.
Wild animals	No wild animals were used in these studies.
Field-collected samples	No field collected samples were used in these studies.
Ethics oversight	Sydney Local Health District (SLHD) Animal Welfare Committee (AWC) and SLHD Institutional Biosafety Committee (IBC) approved SARS-CoV-2 mouse infection experiments.<br>IDX bio-distribution studies in BALB/c nude mice were performed according to the guidelines approved by the Institutional Animal Care and Use Committee (IACUC) of Shanghai Chempartner following the guidance of the Association for Assessment and Accreditation of Laboratory Animal Care.<br>The use of C57BL/6J mice for splenic dendritic cells studies was carried out according to Monash University Animal Ethics committee regulations. The use of TREX1 mutant mice for BMMs was was carried out according Australian National University animal ethics regulations.

Note that full information on the approval of the study protocol must also be provided in the manuscript.
